# Third-Generation Anticancer Photodynamic Therapy Systems Based on Star-like Anionic Polyacrylamide Polymer, Gold Nanoparticles, and Temoporfin Photosensitizer

**DOI:** 10.3390/molecules29102224

**Published:** 2024-05-09

**Authors:** Oleg Yeshchenko, Pavlo Khort, Oles Fedotov, Vasyl Chumachenko, Pavlo Virych, Hunter S. Warren, Brian W. Booth, Valery Bliznyuk, Nataliya Kutsevol

**Affiliations:** 1 Physics Department, Taras Shevchenko National University of Kyiv, 60 Volodymyrska Str., 01601 Kyiv, Ukraine; oleg.yeshchenko@knu.ua (O.Y.);; 2 Chemistry Department, Taras Shevchenko National University of Kyiv, 60 Volodymyrska Str., 01601 Kyiv, Ukraine; vasyl.chumachenko@knu.ua (V.C.); pavlo.virych@knu.ua (P.V.); 3 Department of Bioengineering, Clemson University, Clemson, SC 29634, USAbrbooth@clemson.edu (B.W.B.); 4 Department of Environmental Engineering and Earth Science, Clemson University, Clemson, SC 29634, USA

**Keywords:** polymer nanocarrier, nanosystem, Au nanoparticles, temoporfin, photodynamic therapy

## Abstract

Photodynamic therapy (PDT) is a non-invasive anticancer treatment that uses special photosensitizer molecules (PS) to generate singlet oxygen and other reactive oxygen species (ROS) in a tissue under excitation with red or infrared light. Though the method has been known for decades, it has become more popular recently with the development of new efficient organic dyes and LED light sources. Here we introduce a ternary nanocomposite: water-soluble star-like polymer/gold nanoparticles (AuNP)/temoporfin PS, which can be considered as a third-generation PDT system. AuNPs were synthesized in situ inside the polymer molecules, and the latter were then loaded with PS molecules in an aqueous solution. The applied method of synthesis allows precise control of the size and architecture of polymer nanoparticles as well as the concentration of the components. Dynamic light scattering confirmed the formation of isolated particles (120 nm diameter) with AuNPs and PS molecules incorporated inside the polymer shell. Absorption and photoluminescence spectroscopies revealed optimal concentrations of the components that can simultaneously reduce the side effects of dark toxicity and enhance singlet oxygen generation to increase cancer cell mortality. Here, we report on the optical properties of the system and detailed mechanisms of the observed enhancement of the phototherapeutic effect. Combinations of organic dyes with gold nanoparticles allow significant enhancement of the effect of ROS generation due to surface plasmonic resonance in the latter, while the application of a biocompatible star-like polymer vehicle with a dextran core and anionic polyacrylamide arms allows better local integration of the components and targeted delivery of the PS molecules to cancer cells. In this study, we demonstrate, as proof of concept, a successful application of the developed PDT system for in vitro treatment of triple-negative breast cancer cells under irradiation with a low-power LED lamp (660 nm). We consider the developed nanocomposite to be a promising PDT system for application to other types of cancer.

## 1. Introduction

Photodynamic therapy (PDT) is a modern, non-invasive, rapidly developing, and promising method for treating a wide range of oncological diseases [1]. PDT is based on the interaction of light and a photosensitizer (PS), leading to the formation of cytotoxic reactive oxygen species (ROS). ROS damage cancer cells initiating apoptosis, necrosis, or autophagy [2]. PDT treatment has the advantage of reduced side effects otherwise typically following other methods of radiation or chemotherapy [2,3]. The main limitation of the use of PSs in anticancer treatments is their nonselective accumulation in non-target tissues, poor stability in aqueous solutions, and dark cytotoxicity [2,4]. An ideal PDT system must have several important characteristics including low dark cytotoxicity, selective accumulation in target tissues, optical absorption maxima in the range of the phototherapeutic window (650–850 nm), and final metabolization and excretion from the body [5]. 

The first-generation PS systems were based on hematoporphyrin derivatives, had low chemical purity, and low photogeneration efficiency. The second-generation PSs were represented by pure synthetic compounds having an aromatic macrocycle in their structure (chlorins, phthalocyanines, benzoporphyrins, etc.) and optical absorption in the range of 650–800 nm [6,7]. However, these second-generation PS systems had poor water solubility and formed aggregates under physiological conditions thus reducing the yield of the ROS generation. The experimental and clinical studies of the first- and second-generation PSs demonstrated the need for improvement to achieve better therapeutic effects. Significant advances have been made over the last decade for creating the next generation of photosensitizers; however, these attempts are still mainly in the developmental stage [8,9,10,11,12,13,14,15]. The third-generation PSs rely on the conjugation of second-generation PS with targeting moieties such as amino acids, peptides, or by their encapsulation into nanocarriers (liposomes, micelles, polymers, or nanoparticles) [16,17,18,19]. The encapsulation strategy and application of nanocarriers allows for improvement in biocompatibility and selectivity of the PDT systems as well as enhancement of the phototherapeutic effects [20,21]. Such approaches also improve the stability and hydrophilicity of nanodrugs, reduce side-effects, help maintain a constant rate of PS delivery, ensure targeted delivery of drugs to tumor cells, and limit dark toxicity [22,23]. 

A promising strategy to enhance the anti-tumor efficiency of drugs is the use of metal nanoparticles. Gold nanoparticles (AuNPs) have been studied in the context of various cancer treatments and have demonstrated their potential as an alternative or adjunct to many chemotherapeutic drugs improving therapeutic responses [24]. The effectiveness of AuNPs is based on the surface plasmon resonance effect. This phenomenon is responsible for the characteristic optical properties of gold nanoparticles, such as surface-enhanced Raman scattering (SERS), absorption (SEA), fluorescence (SEF), and photocatalysis [25,26,27,28]. Therefore, a combination of AuNPs and PSs in hybrid nanosystems can enhance ROS generation and cytotoxicity of PDT [29,30]. AuNPs conjugated with drugs or a PS and targeting polymers are effective against various types of cancer [31,32]. Previous studies demonstrated the high impact of plasmon enhancement on ROS photogeneration in applications of nanohybrids containing polymer, photosensitizers, and AuNPs in vitro [33,34,35]. The accumulation of nanosystems based on dextran-graft-polyacrylamide (D-g-PAA) in tumors in mouse xenograft models has been also demonstrated [36]. 

In this study, we synthesized a three-component nanocomposite based on star-like polymer nanocarrier encapsulating gold nanoparticles and the PS temoporfin. Temoporfin is one of a few PS molecules currently approved for medical trials in several European countries [37]. We report on the optical properties of the nanosystem and detailed mechanisms of the observed enhancement of the phototherapeutic effect. We performed physicochemical characterization of the hybrid nanosystem and determined the optimal concentrations of the components that ensure maximum efficiency of the PDT. Additionally, we demonstrate, as proof of concept, successful application of the developed PDT system for in vitro treatment of triple-negative breast cancer cells (TNBC) under illumination with a low-power LED lamp (660 nm).

## 2. Results and Discussion

**Scheme 1:**
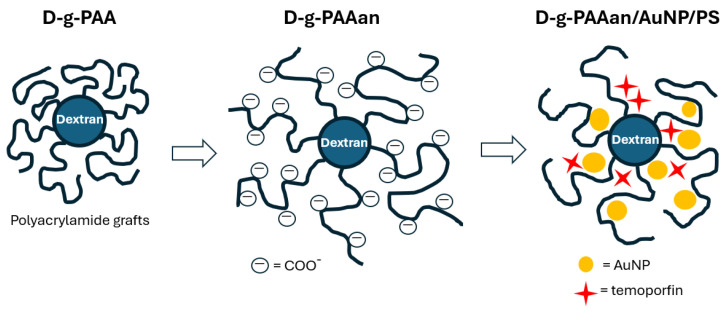
Stages of the PDT system preparation.

The nanocomposite molecular system polymer/NPs/PS was fabricated in three stages. First, a star-like graft copolymer with a dextran core and polyacrylamide arms Dextran-graft-polyacrylamide (D-g-PAA) was synthesized via radical polymerization process and was converted into anionic form (D-g-PAAan) [38,39]. The choice of this copolymer is based on its previously reported efficacy for the design of nanosystems for chemotherapy and PDT [40]. The applied method of synthesis allows for precise control of the conformation of macromolecules through variations in the size of the dextran core and the number and length of grafted arms. This molecular system was used as a matrix for temoporfin and AuNP encapsulation. During the second stage of preparation, AuNPs were synthesized in situ into the polymer matrix in accordance with a previously reported protocol [36]. This method of synthesis allows precise control of the NP diameter and size distribution. The third step of the preparation included mixing of the PS (temoporfin) solution in DMSO with a water dispersion of D-g-PAAan/AuNP particles and was performed just before application of the system to biological systems. Molecular conformation of the system at different stages of the preparation is shown in Scheme 1.

### 2.1. Size Characteristics of Nanohybrids in Aqueous Solution

The physicochemical properties of the system, interaction between the components, and ROS generation are of paramount importance for PDT. Therefore, we performed detailed investigations of the structure and optical properties of the system.

**Figure 1:**
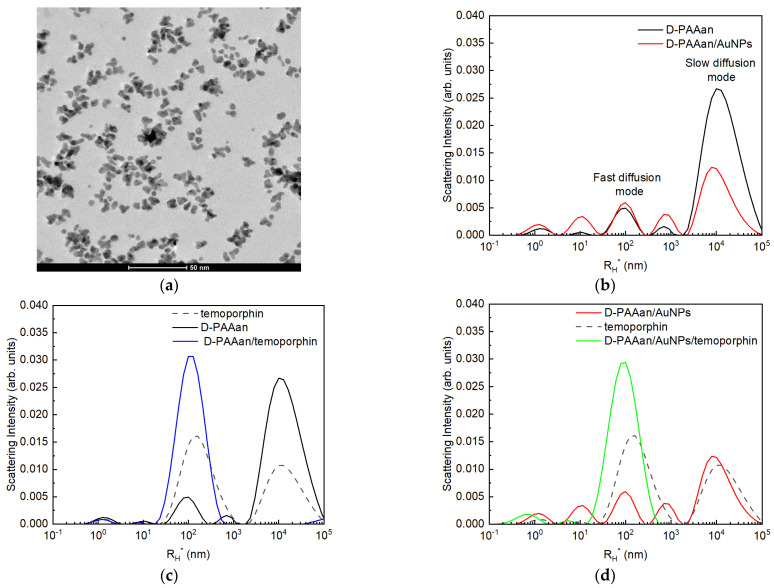
(**a**) TEM image of D-g-PAAan/AuNPs nanohybrids at 20 °C. (**b**–**d**) PSD for D-g-PAAan nanohybrid systems: (**b**) PSD for D-g-PAAan and D-g-PAAan/AuNPs; (**c**) binary temoporfin/polyelectrolyte system and control samples; (**d**) temoporfin/D-g-PAAan/AuNPs and control samples.

A TEM image of the D-g-PAAan/AuNPs nanosystem is shown in Figure 1a. Due to the low contrast of the polymer compared to AuNPs, only metal NPs are visible in the image. However, their spatial location clearly demonstrates the encapsulation of AuNPs into the polymer macromolecules, i.e., the formation of nanohybrids in the pictured samples. Indeed, AuNPs are arranged in groups along the polymer chains of PAAan. The average radius of AuNPs is 3.5 nm. However, given the low contrast of organic molecules (D-g-PAAan and temoporfin) and their aggregates compared to AuNPs, TEM does not provide complete information on the size of the studied nanohybrids. Therefore, size characterization was performed using dynamic light scattering (DLS).

DLS-derived particle size distributions (PSD) (Figure 1b–d) are plotted in hydrodynamic radius R_H_* coordinates. As a quantity which can be found from diffusion coefficients by applying the Einstein–Stokes equation, R_H_* provides an intuitive scale for sample intercomparing. However, we note that R_H_* is not always equivalent to the hydrodynamic radius *R_H_* of non-electrolyte colloid particles or macromolecules.

Polyelectrolyte effects are clearly visible (Figure 1b), where we observe complex multimodal distributions for both D-g-PAAan and D-g-PAAan/AuNPs, instead of a single-peak distribution from an uncharged polymer solution. Namely, there is a fast polyelectrolyte diffusion mode and a slow polyelectrolyte diffusion mode. In brief, the fast diffusion mode corresponds to the diffusion of charged macromolecules and has a notably lower diffusion coefficient and R_H_* compared to uncharged polymers. The slow diffusion mode has a complex and counterintuitive nature, often explained as the diffusion of aggregates. 

Temoporfin cast into water from a DMSO solution clearly forms aggregates, namely moderate aggregates with radii of 145 nm and very large aggregates with radii about 10 μm (Figure 1c, dashed line). This was expected, given that temoporfin is poorly soluble in water.

R_H_* distributions from temoporfin/D-g-PAAan and temoporfin/D-g-PAAan/AuNPs conjugates show a complete disappearance of the slow diffusion mode as well as the disappearance of the previously noted temoporfin aggregates (Figure 1c,d). This observation indicates the quenching of the polyelectrolyte effect and an increase in temoporfin solubility. Size distributions of temoporfin/D-g-PAAan and D-g-PAAan/AuNPs/temoporfin resemble the characteristic distribution curve of uncharged polymers. This effect is a result of polyelectrolyte–temoporfin interaction. AuNPs do not play a significant role in this process. The obtained radii of temoporfin/D-g-PAAan and temoporfin/D-g-PAAan/AuNPs conjugates are 110 nm and 90 nm, respectively. 

Additionally, zeta potentials of D-g-PAAan-based conjugates and control samples (Table 1) reveal a decrease in surface charge for temoporfin/D-g-PAAan and D-g-PAAan/AuNPs/temoporfin compared to bare D-g-PAAan and D-g-PAAan/AuNPs.

### 2.2. Optical Properties of Temoporfin/D-g-PAAan and Temoporfin/D-g-PAAan/AuNPs Nanohybrids

**Figure 2:**
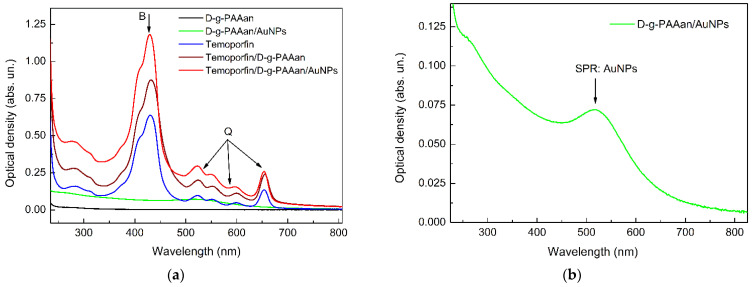

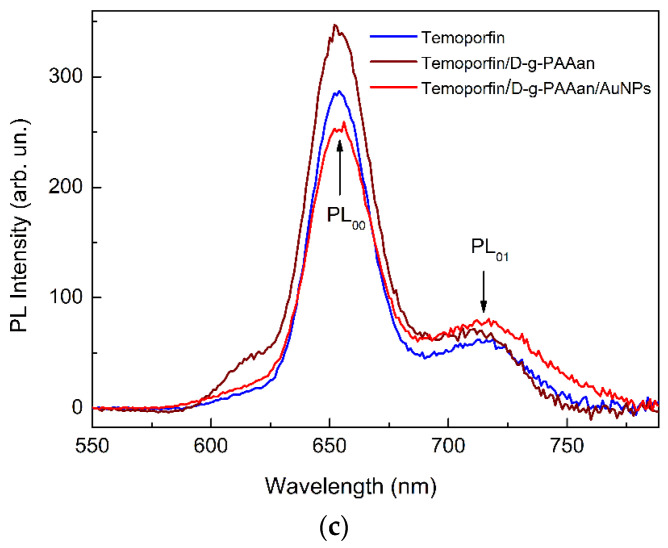
(**a**) Absorption spectra of aqueous solutions of D-g-PAAan and D-g-PAAan/AuNPs, temoporfin mixed with water, and aqueous solutions of D-g-PAAan and D-g-PAAan/AuNPs. (**b**) Absorption spectrum of an aqueous solution of D-g-PAAan/AuNPs. (**c**) PL spectra of temoporfin in water and aqueous solutions of temoporfin/D-g-PAAan and temoporfin/D-g-PAAan/AuNPs under excitation of 423 nm. Concentrations of components: temoporfin—0.72 μg/mL, gold and polymer—7.2 μg/mL.

Absorption spectra of a D-g-PAAan/AuNPs aqueous solution, a mixture of temoporfin in DMSO with water, and aqueous solutions of D-g-PAAan and D-g-PAAan/AuNPs are shown (Figure 2). The absorption spectrum of temoporfin in water (Figure 2a) has features typical for porphyrins. Specifically, the spectrum contains weak long-wavelength (530–620 nm) Q-peaks and an intense short-wavelength (380–440 nm) B (Soret) peak with a characteristic doublet structure [41,42,43,44]. These peaks arise as a result of S_0_ → S_1_ and S_0_ → S_2_ transitions from the π-electron ground state to the 1st and 2nd excited π-electron states of the porphyrin molecule, respectively. The absorption spectrum of the D-g-PAAan/AuNP nanohybrid shows a clear characteristic peak [34] of surface plasmon resonance (SPR) in the AuNPs at 520 nm (Figure 2b). The absorption spectrum of the D-g-PAAan polymer is located in the UV range at wavelengths shorter than 250 nm (Figure 2a), i.e., outside the spectral range relevant to this work consideration.

The characteristic features of the absorption spectrum of bare temoporfin in water and in aqueous solutions of temoporfin/D-g-PAAan and temoporfin/D-g-PAAan/AuNPs are the same. However, these three nanosystems differ significantly by absorption magnitude. The absorption intensity increases when temoporfin is added to the polymer solution, reaching the maximum value in the temoporfin/D-g-PAAan/AuNPs nanosystem. The observed changes in the intensity of absorption when mixing temoporfin with solutions of the polymer and the hybrid nanosystem clearly indicate an association of PS molecules with macromolecules in the D-g-PAAan polymer and the D-g-PAAan/AuNPs nanohybrid systems. 

Temoporfin has low solubility in water. Therefore, when a temoporfin solution in DMSO is added to an aqueous solution of polymer/AuNP, temoporfin precipitation takes place on the polymer particles. The polymer particles act as seeding agents accumulating PS molecules. At the same time, due to an expanded conformation of the star-like macromolecules swollen in water, temoporfin molecules can penetrate the inner space and come in close contact with gold nanoparticles.

Photoluminescence (PL) spectra of temoporfin in water, the temoporfin/D-g-PAAan, and temoporfin/D-g-PAAan/Au NP nanosystems are shown (Figure 2c). The PL spectrum of temoporfin in water has a structure typical for porphyrins [42,44]. To be exact, the spectrum contains two peaks. These are short-wave (653 nm) PL_00_ and long-wave (715 nm) PL_01_ peaks. These peaks originate from S_1_(0) → S_0_(0) and S_1_(0) → S_0_(1) radiative transitions from the ground vibrational level of the 1st excited π-electron state to the ground and 1st excited vibrational levels of the ground π-electron state of the PS molecule, respectively. In addition to the intense PL_00_ and PL_01_ peaks, there is also a weak peak at 618 nm in the PL spectrum. This is probably due to radiative transitions in the phenyl group bound to the porphyrin ring. The intensity of the peak at 615 nm of bare temoporfin is quite low but increases when temoporfin is bound to the polymer. This is an additional argument in favor of our conclusion about the binding of temoporfin molecules to the polymer, which is discussed below.

The PL spectrum of temoporfin significantly changes when temoporfin is mixed with polymer solutions or solutions of nanohybrid comprised of polymer with gold nanoparticles (Figure 2c). Mixing temoporfin with an aqueous solution of the polymer D-g-PAAan leads to an increase in the intensity of the PL spectrum compared to the intensity of the spectrum of bare temoporfin in water. This effect is analogous to the increase in the intensity of light absorption by temoporfin when mixed with a polymer solution. At the same time, mixing temoporfin with a solution of nanohybrid D-g-PAAan/AuNPs leads to the opposite effect: the intensity of the spectrum decreases compared not only to the temoporfin/D-g-PAAan system but also to bare temoporfin in water. The observed PL quenching when adding PS to the nanohybrid solution containing gold NPs is observed only at high concentrations of the components, while the reverse effect of PL intensity growth is observed at low concentrations. Concentration effects in the PL spectrum and their physical mechanisms are discussed below. In addition to changes in the total PL intensity, there is also a relative change in the intensities of PL_00_ and PL_01_ peaks in systems containing AuNPs and in which AuNPs are absent. Accordingly, based on PL data, we concluded that temoporfin molecules interact (bind) with D-g-PAAan and D-g-PAAan/AuNPs macromolecules, and this interaction is different in the presence and absence of AuNPs.

The binding of temoporfin molecules to D-g-PAAan and D-g-PAAan/AuNPs macromolecules was verified directly by measuring the PL anisotropy coefficient *r*, which characterizes the degree of freedom of molecules possess during their motion. PL anisotropy for temoporfin in water is 5%. Mixing temoporfin with an aqueous solution of D-g-PAAan leads to an increase in the PL anisotropy (10%), which indicates the binding of temoporfin molecules to polymer macromolecules. The value of *r* for temoporfin in an aqueous solution of temoporfin/D-g-PAAan/AuNPs is 14%, indicating that temoporfin molecules bind to the hybrid macromolecules that contain AuNPs. It is worth noting that the value of the PL anisotropy does not depend on the concentration of the components in the entire studied concentration range. An additional argument in favor of the conclusion regarding the binding of temoporfin molecules to the polymer and hybrid macromolecules is the increase in the intensity of the weak 615 nm PL peak, which occurs when temoporfin is mixed with D-g-PAAan and D-g-PAAan/AuNPs solutions, that is noted above.

**Figure 3:**
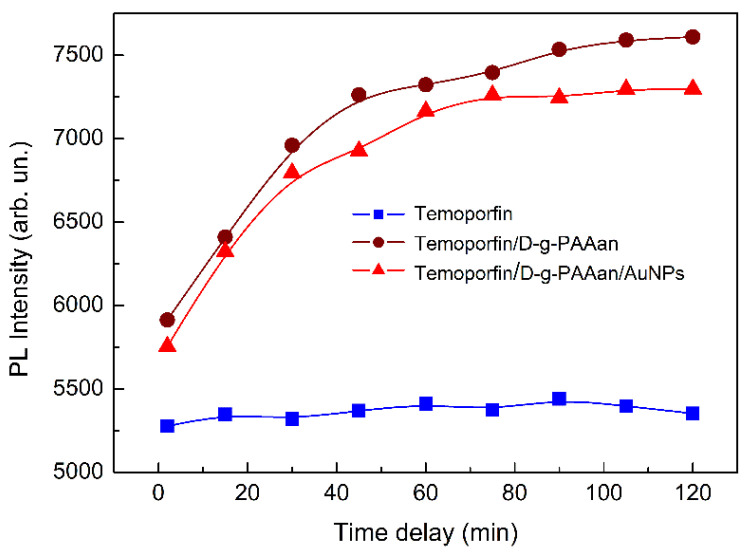
Dependence of intensity of temoporfin PL on the time elapsed after mixing with water and aqueous solutions of D-g-PAAan and D-g-PAAan/AuNPs. Concentrations: temoporfin—0.72 μg/mL, D-g-PAAan and Au—7.2 μg/mL.

The behavior of the total PL intensity of temoporfin as a function of time after mixing temoporfin in DMSO with water and aqueous solutions of D-g-PAAan and D-g-PAAan/AuNPs was investigated (Figure 3). The PL intensity of temoporfin in water does not depend on the time after mixing. However, the temporal behavior of the PL intensity of temoporfin in the composition of temoporfin/D-g-PAAan and temoporfin/D-g-PAAan/AuNPs nanosystems is fundamentally different; specifically, during the first 30 min the intensity increases sharply, the growth rate then decreases, and after 90 min, the dependence remains constant. During the first day after mixing, the PL intensity remains constant. Starting from the second day, the PL intensity gradually decreases. The increase in the PL intensity of temoporfin in the composition of the temoporfin/D-g-PAAan and temoporfin/D-g-PAAan/AuNPs systems proves the coordination of temoporfin molecules with the macromolecules of the polymer and the Au containing nanohybrids. As evidenced by DLS data, the binding of PS molecules to macromolecules of D-g-PAAan and D-g-PAAan/AuNPs leads to the destruction of the aggregates of temoporfin molecules, which causes an increase in PL intensity. Quenching of PL, which is observed starting from the second day after mixing, probably occurs due to the oxidation of temoporfin.

Studying the influence of changes in the concentration of PS, polymer, and AuNPs on the optical properties of the investigated nanosystems is important for understanding the impact of the interactions of temoporfin with polymer and AuNPs on electronic processes in temoporfin molecules in hybrid nanosystems. The changes in the concentrations of PS molecules and metal NPs cause changes in the mean PS molecule–AuNP distances inside the polymer macromolecules. The distance changes influence the strength of coupling of metal NP and PS molecules [40,45,46,47,48,49]. Also, the coupling strength depends strongly on the spectral overlap of SPR in metal NPs and the electronic energy spectrum of the PS molecules. The coupling is stronger at shorter distances and higher overlap. Since temoporfin molecules and AuNPs are closely located inside the hybrid macromolecule and there is a significant overlap of the AuNP SPR absorption band with the absorption and PL spectra of temoporfin molecule, it is reasonable to expect the strong coupling of AuNPs with temoporfin molecules in temoporfin/D-g-PAAan/AuNPs nanohybrids. The coupling occurs through two physical mechanisms. These mechanisms are plasmon enhancement and non-radiative energy transfer between the PS molecule and metal NP (Förster resonant energy transfer, FRET).

The concentration dependences of the total optical density and total PL intensity of temoporfin in water and aqueous solutions of D-g-PAAan and D-g-PAAan/AuNPs were determined (Table 2). An increase in the concentration of PS leads to an increase in the optical density of temoporfin in water (Table 2), which is quite expected. At the same time, in the temoporfin/D-g-PAAan system, the absorption concentration increases faster than bare temoporfin. This is due to the destruction of PS aggregates in the presence of the polymer. In the triple temoporfin/D-g-PAAan/AuNPs nanosystem, the increase in absorption occurs even faster than in the temoporfin/D-g-PAAan system. This is due to the plasmon enhancement of light absorption by temoporfin molecules located near the gold NPs in the nanohybrid macromolecule.

The effect of changing the concentration of the polymer and AuNPs on the total PL intensity of temoporfin in the temoporfin/D-g-PAAan and temoporfin/D-g-PAAan/AuNPs nanosystems was investigated (Table 2). In general, an increase in the concentration of PS with a simultaneous increase in the concentrations of polymer and gold leads to an increase in the PL intensity of temoporfin. However, these dependencies are more complex compared to absorption. First, at minimal concentrations of PS, polymer, and gold, the PL intensity is minimal for temoporfin in water, intermediate for the temoporfin/D-g-PAAan system, and maximal for the triple temoporfin/D-g-PAAan/AuNPs nanosystem. Indeed, at the minimal concentration, the distance between PS molecules and gold NPs is maximal, which is sufficient for the existence of plasmonic enhancement of PL, but it is too large for the efficient action of FRET between the PS molecules and AuNPs, which quenches PL. Second, an increase in the concentration of temoporfin in water leads to a sublinear increase in the intensity of PL, which is due to the strengthening of aggregation in the system of hydrophobic molecules of the PS. At higher concentrations, the distance between PS molecules and AuNPs decreases leading to increased FRET. This results in a slowdown in the concentration growth of PL intensity for the temoporfin/D-g-PAAan/AuNPs system. A further increase in concentration leads to a further decrease in the distance between PS molecules and gold NPs, and as a result, a significant predominance of FRET over plasmon enhancement is observed. At the highest concentration, the PL intensity of the PS in the temoporfin/D-g-PAAan/AuNPs system is the lowest (Table 2). In turn, an increase in the concentration of the polymer leads to a faster increase in PL intensity compared to the case of bare temoporfin in water, which is caused, as mentioned above, by the destruction of PS aggregates in the presence of the polymer. Since the initial electronic state of the PS molecule is the ground (unexcited) state during absorption transitions, these transitions are not affected by FRET but only by plasmon enhancement.

Thus, the PL intensity of PS molecules is determined by two competing physical mechanisms. The first is the plasmon enhancement, which is stronger when the distance between the molecules and the gold NPs is smaller [40,45,46,47,48,49,50]. The strength of the plasmon field depends on the distance to the metal NP as $E_{sp}\propto R^{-3}$ [49,51]. The second mechanism is Förster dipole–dipole resonance non-radiative energy transfer (FRET), in which the energy of an excited donor (PS molecule) can be non-radiatively transferred to an acceptor (metal NPs) [40,45,46,47]. FRET leads to PL quenching. The PL quenching rate due to FRET depends on the donor–acceptor distance as $E_{FRET}\propto R^{-6}$. This effectively limits FRET to below 10 nm. As a result of the competition between PL quenching due to FRET and PL plasmon enhancement, there is an optimal distance between the metal NPs and the PS molecules (about 10 nm) at which the PL intensity is the highest. At distances less than 10 nm, a small decrease in the distance leads to a strong decrease in PL intensity, i.e., to PL quenching. At distances greater than 10 nm, a decrease in the distance leads to an increase in PL intensity, i.e., to PL enhancement.

Therefore, at low concentrations of AuNPs (0.72 μg/mL), the distance between temoporfin molecules and AuNPs is too large for FRET. An increase in the concentration of AuNPs and PS molecules leads to a decrease in the distance between molecules and NPs, which causes an increase in the PL plasmon enhancement. At higher concentrations of gold (7.2 μg/mL), the distance between the temoporforin molecules and the NPs becomes small so that the FRET process is triggered leading to PL quenching when the gold concentration increases. Thus, we conclude that there is an optimal concentration of AuNPs and temoporfin molecules that provides the greatest plasmon enhancement of various electronic processes involving the photosensitizer temoporfin, in particular the photogeneration of singlet oxygen.

### 2.3. ROS Generation

A study of ROS generation under photodynamic therapy conditions for D-g-PAAan/AuNPs/Temoporfin systems was carried out. The aim was to exclude cellular mechanisms of ROS generation, for example, mitochondria damage by AuNPs [51].

**Figure 4:**
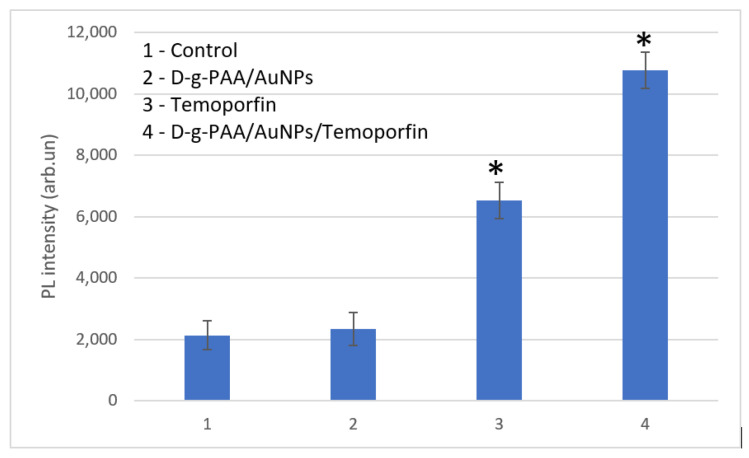
ROS level for saline (control), D-g-PAAan/AuNPs (72 μg/mL), temoporfin (7.2 μg/mL) and D-g-PAAan/AuNPs/temoporfin (72 μg/mL D-g-PAAan/AuNPs, 7.2 μg/mL temoporfin) after irradiation with 660 nm light. Error bars indicate the standard deviation; *n* = 6, Kruskal–Wallis test, * *p* < 0.05.

No statistically significant differences in ROS levels were found after 660 nm light irradiation of saline and D-g-PAAan/AuNPs (Figure 4). ROS levels increased ~3X in a temoporfin solution and ~5X for the triple nanocomposite D-g-PAAan/AuNPs/temoporfin solution.

The results prove the degradation of the photosensitizer and generation of free radicals after the 660 nm light irradiation. The conjugation of AuNPs and temoporfin in the D-g-PAAan polymer matrix enhances ROS formation. Thus, energy is exchanged between the photosensitizer and the nanoparticles with an increase in ROS generation.

### 2.4. Photodynamic Therapy In Vitro

**Figure 5:**
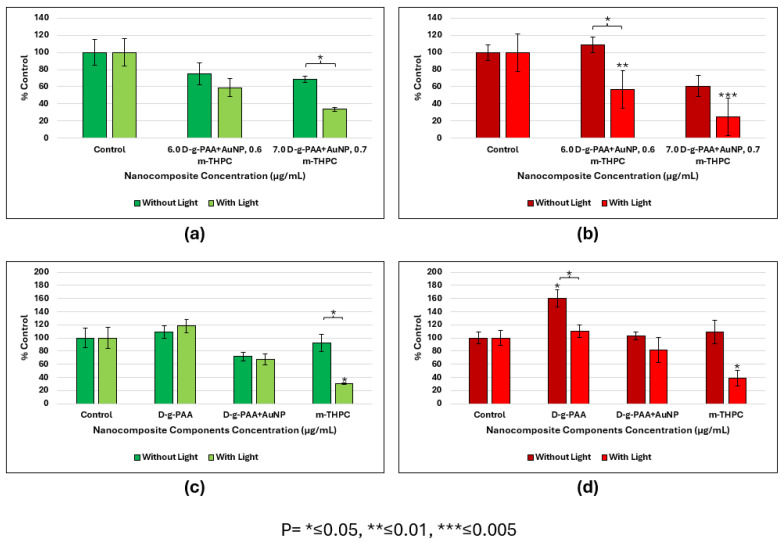
Cell counts 24 h after PDT treatment with select nanocomposite concentrations. (**a**) Human breast epithelial cells that did not receive light are denoted with dark green, and human breast epithelial cells that did receive light are denoted with light green *(n* = 3), (**b**) TNBC cells that did not receive light are denoted with dark red, and cells that did receive light are denoted with light red (*n* = 6). (**c**) Human breast epithelial cells and (**d**) TNBC cells were exposed to the individual components of the nanocomposite with and without light treatments. Error bars indicate the standard deviation. * *p* < 0.05, ** *p* < 0.01, *** *p* < 0.005.

The potency of the nanocomposite comprised of polymer, AuNPs, and temoporfin was tested to deduce the efficacy of the nanocomposite to kill triple-negative breast cancer (TNBC) cells. The IC_50_ of the nanocomposite was determined to be 4 μg/mL D-g-PAAan/AuNPs with 0.4 μg/mL temoporfin. The cell counts of MDA-MB-231 TNBC cells declined significantly with nanocomposite applications (Figure 5b), while no significant decrease in cell numbers was observed when normal breast epithelial cells received identical treatments (Figure 5a). No significant changes in cell numbers were observed in normal breast epithelial cell cultures that were exposed to the individual components of the nanocomposite with or without red light exposure (Figure 5c). When TNBC cells were treated with individual components only the m-THPC resulted in a reduction in cell number following red light exposure (Figure 5d). 

**Figure 6:**
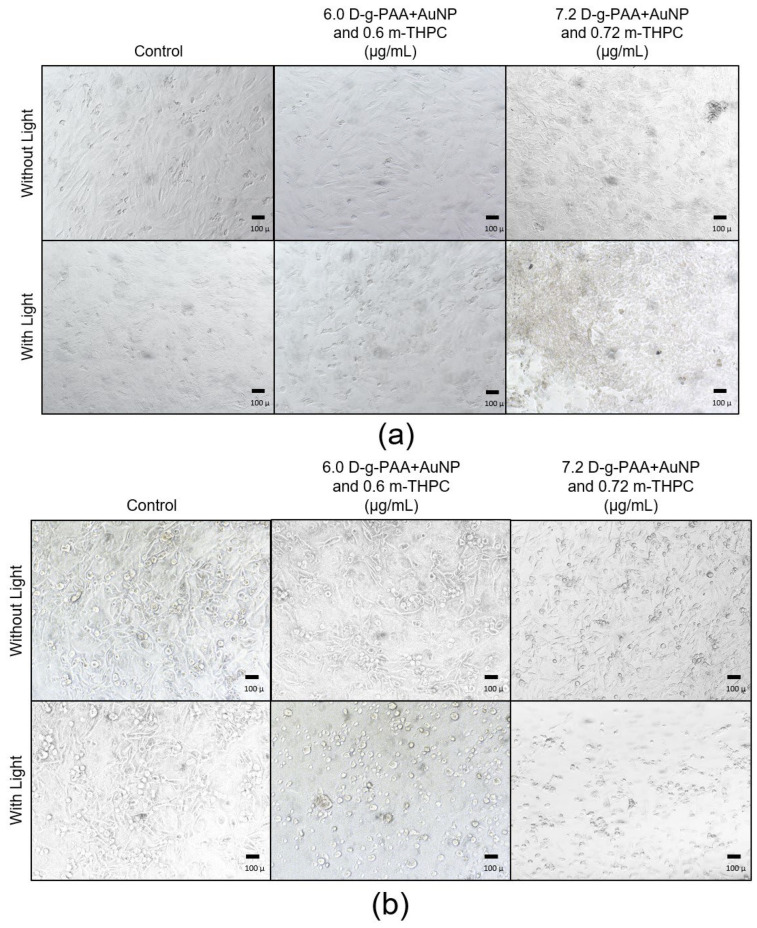
Cell morphology 24 h after PDT treatment with select nanocomposite concentrations. Different PDT treatment concentrations for: (**a**) normal breast epithelial cells and (**b**) TNBC cells. Those that received light are on top, and those that did not receive light are on the bottom. Scale bars = 100 μm.

The combination of nanocomposite and light treatment induced cellular morphology changes, suggesting the induction of apoptosis in the breast cancer cells (Figure 6a). No similar shape changes were observed in the breast epithelial cells after exposure (Figure 6b). At both concentrations tested, the breast cancer cell numbers were significantly decreased. Red light exposure induced a significant reduction in cancer cell number compared to the samples that did not receive light treatments (Figure 5b and Figure 6b). This indicates that red light treatment activates the PS and AuNPs resulting in increased cell death. The light treatments had minimal effect on the breast epithelial cells that received the nanocomposite.

## 3. Materials and Methods

### 3.1. Materials

All chemicals for the synthesis of polymer-nanocarriers and metal containing polymer nanosystems were purchased from Merck (Munich, Germany) and used without further purification, except where explicitly mentioned.

### 3.2. Polymer Nanocarrier

Star-like copolymer D-g-PAAan was used as a nanocarrier and a matrix for nanosystem preparation. D-g-PAA in non-ionic form was synthesized via the radical graft polymerization method reported previously [38]. PAA chains were grafted on certified dextran with molecular weights M_W_ = 7 × 10^4^ g/mol. The number of PAA grafts was controlled by the molar ratio of the added initiator to dextran to obtain a copolymer with 5 theoretical grafts. The molecular parameters of this copolymer were M_W_ = 2.15 × 10^6^ g/mol, Rz = 85 nm, and M_W_/Mn = 1.72. Then, the D-g-PAA sample was saponified to obtain the copolymer in anionic form. Alkaline hydrolysis of D-g-PAA resulted in the transformation of amide groups of PAA chains to carboxylate; this is one of the peculiarities of the internal structure of copolymer in anionic form (D-g-PAAan, Scheme 1) as previously reported [39]. Alkaline hydrolysis was not accompanied by destruction or cross-linking of macromolecules. The conversion degree of amide groups into carboxylate groups with 30 min of hydrolysis calculated from the potentiometric titration data was 36%. 

### 3.3. Nanosystem Preparation

D-g-PAAan/AuNPs: 1 g of D-g-PAAan was dissolved in 100 mL of water and kept for 24 h to achieve full dissolution. A total of 1 mL of aqueous HAuCl_4_ 10^−2^ M was then added to 9 mL D-g-PAAan. After 20 min of stirring, 0.4 mL of 10^−1^ M of fresh NaBH_4_ solution was added and stirred for 20 min.

Temoporfin/D-g-PAAan/AuNPs: Appropriate volumes of 3 × 10^−3^ mg/mL temoporfin solution in DMSO were added to the stock D-g-PAAan/AuNPs or D-g-PAAan solution to generate the desired concentrations and stirred for 20 min. The applied concentration of DMSO was shown to have negligible cytotoxicity (Figure S1). The procedure was performed just before application of the nanosystem to biological studies. 

### 3.4. Optical Characterization

Absorption and PL spectra were recorded using a Cary 60 UV-VIS spectrophotometer (Agilent Technologies Inc., Santa Clara, CA, USA) and a Shimadzu RF-6000 spectrofluorophotometer (Shimadzu Corp., Kyoto, Japan), respectively. PL was excited by a wavelength of 423 nm. The spectra were measured at room temperature for two hours after mixing the temoporfin solution in DMSO with water and aqueous solutions of D-g-PAAan and D-g-PAAan/AuNPs at 15 min intervals.

Polarization measurements of PL spectra were carried out to determine the degree of PL anisotropy. PL anisotropy was determined as the following:

$$r=\frac{I_{VV}-{GI}_{VH}}{I_{VV}+{2GI}_{VH}}$$

where *I_iJ_* is the total PL intensity, *ij* indices denote the orientation of the polarizers before and after the sample, respectively (*V*—vertical and *H*—horizontal orientation), and $G=I_{HV}/I_{HH}$ is the grating factor [52].

### 3.5. Dynamic Light Scattering Characterization

Dynamic light scattering (DLS) studies of samples were performed using a NanoBrook Omni particle size analyzer (Brookhaven Instruments, Holtsville, NY, USA) equipped with a 532 nm laser. The scattered light was measured at an angle of 173° (backscattering). The samples were kept at 25 °C for 5 min before measuring to achieve equilibrium. Fifteen correlation curves for each sample were processed by the regularized singular-value decomposition (SVD) algorithm. As a result, we obtained the hydrodynamic particle size distribution (PSD) for the colloidal particles in the studied samples.

### 3.6. ROS Detection In Situ

Temoporfin (7.2 μg/mL), D-g-PAAan/AuNPs (72 μg/mL), and D-g-PAAan/AuNPs/temoporfin solutions (7.2 μg/mL temoporfin + 72 μg/mL D-g-PAAan/AuNPs) in saline were prepared. ROS was measured using a modified method by Daliang et al. [53]. Three milliliters of each solution were added to 2′,7′-dichlorofluorescein (DCFH, Sigma-Aldrich, Burlington, MA, USA) for ROS detection. The final DCFH concentration was 15 × 10^−6^ M. The solutions were irradiated with 660 nm light. The light power was 100 mJ/s, and the irradiation dose was 30 J/mL. Fluorescence measurements were collected immediately after the end of light irradiation. A Shimadzu RF-6000 spectrofluorophotometer with LabSolutions RF (ver. 1.11) software was used. Fluorescence was registered at 525 nm with 488 nm excitation. The fluorescence of the samples was evaluated in arbitrary units. All samples were in the same conditions during the experiment. This study was repeated six times.

### 3.7. In Vitro Cell Culture

The human TNBC line MDA-MB-231 and the human breast epithelial cell line MCF10A were used in this study (ATCC, Manassas, VA, USA). The MDA-MB-231 cell line was maintained in Dulbecco’s Modified Eagle Medium (DMEM, Corning, NY, USA) supplemented with 10% fetal bovine serum (FBS; Corning, NY, USA) and 1% antibiotic/antimycotic (AA; ThermoFisher, Waltham, MA, USA). The MCF10A cell line was grown in DMEM enhanced with 10% FBS, 1% AA, and MEGM SingleQuots containing insulin, hydrocortisone, GA-1000, hEGF, and BPE (Lonza, Walkersville, MD, USA). All the cell cultures were grown at 37 °C in a humidified atmosphere of 5% CO_2_.

### 3.8. Photodynamic Therapy Protocol

The complete procedures for the PDT treatment were performed in the dark to prevent an unwanted reaction of the light-sensitive treatment concentrations with the atmospheric light. MDA-MB-231 and MCF10A cell lines were seeded in well plates and grown to 70% confluence. The culture medium was removed, and the cells were washed with PBS. The different doses of nanocomposite treatment concentrations were then added to the wells. The control wells only received DMEM. The cultures were then incubated for 90 min with the nanocomposite. At the end of the incubation period, the treatment medium was removed from the plates. The wells were washed three times with PBS to avoid interference of the phenol red in the DMEM with the wavelengths of the red-light source. Subsequently, the plate was exposed to light with the third wash of PBS on the wells. The plates were placed in a cell culture hood 15 cm below a low-power red light for 10 min without a lid. As a light source, a 660 nm Deep Red LED Light Bulb for red light therapy (ABI-A Brighter Idea, Inc., Rockville, MD, USA) with a power density of 93.6 mW/cm^2^ and light dose of up to 28.1 J/cm^2^ was used. The red light was covered with aluminum foil to reduce light scatter and to expose the plate to the full intensity of the red light. After this step, the PBS was removed and replaced with the culture medium of the cells. For the experiments to evaluate the toxicity of the nanocomposite in the dark, the cells of a second plate were treated under the same conditions but were not exposed to red light. The plates not exposed to light were filled with the culture medium right after the third wash with PBS. The plates were then kept in the incubator for 24 h before the experiments detailed below were performed.

### 3.9. Cell Counts and Morphology

After PDT protocols with varying concentrations and constituents were implemented, the cell morphology was imaged after 24 h with a Zeiss Axiovert 40 CFL inverted microscope and collected with a Zeiss AxioCam camera and AxioVision 4.8 software (Pleasanton, CA, USA). Cell counts were performed manually using a hemocytometer after the cells were washed with PBS, detached with 0.05% trypsin (Corning, Manassas, VA, USA) and neutralized with cell culture media. 

### 3.10. Statistical Analysis and Figures

Statistical analyses for samples with one variance were performed using pair of two samples for a mean value, and statistical analyses for samples with more than one variance were performed with single-factor analysis of variance. Tests and plots were generated using Excel (Microsoft, Washington, DC, USA, https://www.microsoft.com/en-us/microsoft-365/excel, accessed on 6 March 2024). Experiments had a minimum of *n* = 3 per group. The Kruskal–Wallis test (*p* < 0.05, *n* = 6) was used to analyze fluorescence when measuring ROS levels. Cell counts were analyzed using a one-way ANOVA for comparisons between the treatment groups, and student’s *t*-test was used for comparisons between the illuminated and control treatment groups. The date reported shows the mean value ± the standard deviation. A *p*-value ≤ 0.5 was considered significant. 

## 4. Conclusions

We have demonstrated the efficiency of a ternary PDT system comprising a dextran/polyacrylamide star-like polymer with incorporated temoporfin and gold nanoparticles in the generation of cytotoxic reactive oxygen species under 660 nm LED light excitation. Detailed studies of the system’s optical properties and structure using dynamic light scattering and absorption/emission spectroscopy revealed peculiarities of the excitation mechanism responsible for PDT effects and allowed optimization of the composition to achieve higher therapeutic effects. We demonstrated the applicability and effectiveness of the system in PDT cancer treatment by comparative in vitro studies of human triple-negative breast cancer cells and human breast epithelial cells. Though this study gives a proof of concept only, the developed system is ready for in vitro studies and can be modified for application to a broad variety of cancer types.

## Figures and Tables

**Table 1:** Zeta potentials of studied PDT samples.

Sample	Zeta-Potential, mV
D-g-PAAan	−70.1
D-g-PAAan/AuNPs	−45.8
Temoporfin	−2.1
D-g-PAAan/Temoporfin	−19.04
D-g-PAAan/AuNPs/Temoporfin	−14.55

**Table 2:** The total optical density and total PL intensity of temoporfin in water and aqueous solutions of temoporfin/D-g-PAAan and temoporfin/D-g-PAAan/AuNPs nanosystems. Optical density and PL intensity are normalized to the corresponding values for temoporfin in water at its minimal concentration of 0.072 μg/mL.

Sample	Normalized Absorption (Total Optical Density), rel. un			Normalized Total PL Intensity, rel. un.		
	1	2	3	1	2	3
0.072 μg/mL (Temoporfin), 0.72 μg/mL (D-g-PAAan/Au)	1	1.38	1.58	1	1.24	1.51
0.72 μg/mL (Temoporfin), 7.2 μg/mL (D-g-PAAan/Au)	4.32	10.11	11.66	2.60	3.53	3.28
7.2 μg/mL (Temoporfin), 72 μg/mL (D-g-PAAan/Au)	45.68	80.45	95.24	6.18	7.22	5.68

1—Temoporfin; 2—Temoporfin/D-g-PAAan; 3—Temoporfin/D-g-PAAan/AuNPs.

## Data Availability

Data are contained within the article.

## Supplementary Materials

The following supporting information can be downloaded at: https://www.mdpi.com/article/10.3390/molecules29102224/s1, Figure S1: Cytotoxicity of DMSO depending on the concentration for MDA-MB-231 cells.
