# Supplementary figures and images for: Third-Generation Anticancer Photodynamic Therapy Systems Based on Star-like Anionic Polyacrylamide Polymer, Gold Nanoparticles, and Temoporfin Photosensitizer
Source: Molecules. 2024 May 9;29(10):2224. doi: 10.3390/molecules29102224 (PMC11123958; doi:10.3390/molecules29102224)

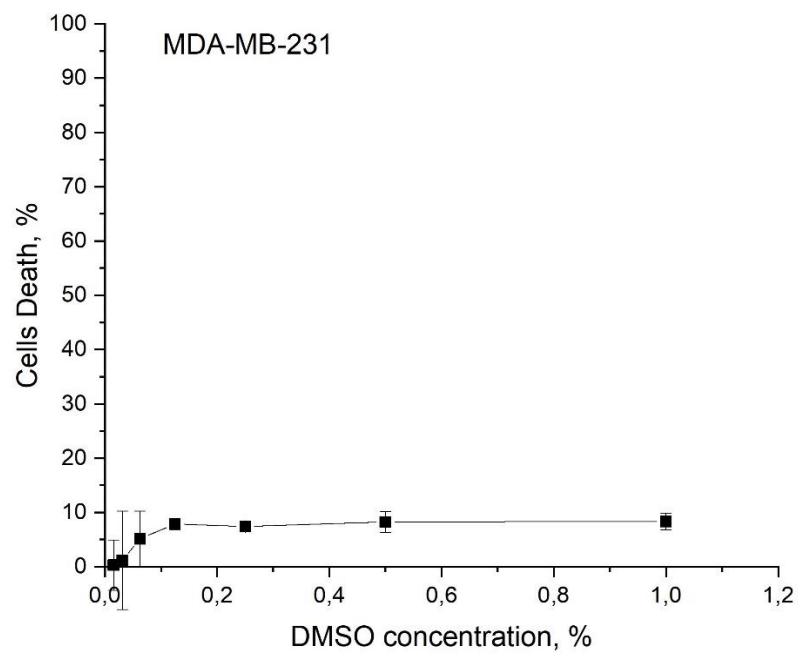

**Figure S1.** Cytotoxicity of DMSO depending on the concentration for MDA-MB-231 cells.

Supplement: Supplementary file 1 [file molecules-29-02224-s001.zip › molecules-2928702-supplementary.pdf]
